# Neutrophil extracellular traps in breast cancer and beyond: current perspectives on NET stimuli, thrombosis and metastasis, and clinical utility for diagnosis and treatment

**DOI:** 10.1186/s13058-019-1237-6

**Published:** 2019-12-18

**Authors:** Hunter T. Snoderly, Brian A. Boone, Margaret F. Bennewitz

**Affiliations:** 1 Department of Chemical and Biomedical Engineering, West Virginia University, 1306 Evansdale Drive, ESB 521, Morgantown, WV 26506 USA; 2 Department of Surgery, West Virginia University, Morgantown, WV 26506 USA

**Keywords:** Neutrophil extracellular traps, Breast cancer, Metastasis, Venous thromboembolism

## Abstract

The formation of neutrophil extracellular traps (NETs), known as NETosis, was first observed as a novel immune response to bacterial infection, but has since been found to occur abnormally in a variety of other inflammatory disease states including cancer. Breast cancer is the most commonly diagnosed malignancy in women. In breast cancer, NETosis has been linked to increased disease progression, metastasis, and complications such as venous thromboembolism. NET-targeted therapies have shown success in preclinical cancer models and may prove valuable clinical targets in slowing or halting tumor progression in breast cancer patients. We will briefly outline the mechanisms by which NETs may form in the tumor microenvironment and circulation, including the crosstalk between neutrophils, tumor cells, endothelial cells, and platelets as well as the role of cancer-associated extracellular vesicles in modulating neutrophil behavior and NET extrusion. The prognostic implications of cancer-associated NETosis will be explored in addition to development of novel therapeutics aimed at targeting NET interactions to improve outcomes in patients with breast cancer.

## Background

Neutrophils are the most abundant type of white blood cells in the circulation and are often considered the frontline defenders in innate immunity [1]. These leukocytes were only recently observed to be capable of a novel immune response in which they expel their DNA and intracellular contents in a web-like structure known as a neutrophil extracellular trap (NET). NETs form when activated neutrophils release DNA, histones, and granular content, exposing antimicrobial and proinflammatory proteins [2]. NETosis occurs as specific proteases are translocated into the neutrophil nucleus, which causes their chromatin to decondense through citrullination. These loosely networked strands are then ultimately expelled from the cell, rupturing it or leaving the membrane intact. Subsequent membrane integrity depends on the nature of the stimulus provoking NETosis [3]. NETs were first observed as a response to bacterial infection, as histones, and released neutrophil granular content have antimicrobial properties and the fibrous NET structure can physically entrap and kill bacteria [2]. However, NETs have since been associated with sterile inflammation in a variety of disease states, including gout, cystic fibrosis, type 1 diabetes, rheumatoid arthritis, preeclampsia, and others [4–9]. NETs have also been associated with tumor cell proliferation and metastasis [10–16], cancer-related thrombosis [17–21], and primary tumor growth [22, 23].

In this review, we will focus on the role of NETs primarily in breast cancer. Globally, breast cancer accounted for around 11.6% of new cancer diagnoses in 2018 and was estimated to be responsible for more than 6% of all cancer deaths [24]. Current evidence suggests that NET production in cancer involves a complex interplay between a variety of cells and blood components, including platelets, leukocytes, pioneering metastatic tumor cells, and the primary tumor site itself [10, 19, 21, 25–28]. NETs promote the progression of an inflammatory microenvironment, which develops a positive feedback loop: NETs released into the circulation damage endothelial cells, which promotes further inflammation, causing activation of platelets and other neutrophils which can cause further NET release. Platelet activation caused by NETs can also promote several negative outcomes associated with late-stage metastatic breast cancer, including venous thromboembolism (VTE) [29]. This review will discuss both established and potential stimuli that promote oncogenic NETosis, both on a molecular level and in terms of interactions between neutrophils, other blood components in cancer-affected organisms, and tumor cells themselves. We will also discuss the consequences of NETosis, especially as it relates to breast cancer progression. Finally, the use of NETs as potential diagnostic biomarkers and/or clinical therapeutic targets in cancer will be discussed.

### Cellular and molecular stimulants of NETosis

#### Pro-NETotic stimuli and neutrophil components required for NETosis

Several potential pro-NETotic stimuli relevant to cancer progression are listed in Table 1. The most classical and potent stimuli provoking NET formation are products of bacterial infection, such as lipopolysaccharide (LPS), or non-endogenous inflammatory pathway activators such as phorbol 12-myristate 13-acetate (PMA) [2]. LPS and PMA promote NETosis through production of reactive oxygen species (ROS) in which oxygen is transformed into damaging superoxide radicals and secondary oxidants. ROS are key to cancer and inflammatory signaling as well as neutrophil behavior modulation [45, 46]. The inflammatory state associated with cancer also may provoke systemic oxidative stress. The presence of higher levels of NETosis observed in many cancers may (at least partially) be attributed directly to tumor cells, as well as indirectly via ROS generation by other cells and granules activated by downstream effects of tumor released factors. It has been shown that PMA provokes NETosis through activation of p38 MAPK via NADPH oxidase generation of ROS [32]; thus, endogenous stimulants may follow similar pathways. Interestingly, p38 activation has also been shown to promote breast cancer cell survival and proliferation and has been linked to poor clinical outcomes in humans [47, 48].

**Table 1:** Key NET stimuli involved in cancer progression. References are annotated to indicate whether NETotic effect has been shown in human (H) neutrophils, mouse (M) neutrophils, or both (HM)

Stimulus/model:	Relevance to cancer progression:	Origin:
LPS [2, 30, 31]^HM^	May simulate response to infection; repeated intranasal dosage in mice activated dormant cancer cells and enhanced metastatic proliferation	Gram-negative bacteria
PMA [2, 32]^H^	N/A	Synthetic/pharmaceutical
Platelet-activating factor [19]^M^	Promotes tumor cell proliferation, neovascularization, and immunosuppressive phenotype	Leukocyte, platelet, and endothelial secretion in inflammation
HMGB1 [14, 25, 33]^HM^	Associates with existing NETs; role in platelet and neutrophil activation; synergizes with LPS and thus may exacerbate response to infection	Leukocyte and platelet secretion in inflammation; expressed in some tumors; released during cell death
IL-8 [5, 34, 35]^H^	Drives neutrophilia; positive correlation with poor outcome in women with breast cancer	Expressed in some tumors; released from activated endothelial cells
G-CSF [19, 36, 37]^M^	Drives neutrophilia; positive correlation with metastasis; potentiates extracellular vesicle driven NETosis	Expressed in some tumors
PAD4 [38–40]^HM^	Catalyzes histone citrullination; inhibition prevents NETosis in most circumstances	Neutrophils; expressed in some tumors
P-selectin [41]^M^	Facilitates neutrophil motility; drives platelet-neutrophil aggregation	Endothelial cells; platelets
TF [42–44]^H^	Activates platelets which activate neutrophils and causes NETosis, potentially through multiple pathways	Secreted during NETosis; expressed in some tumors; contained in tumor EVs
Tumor EVs [21]^M^	May influence neutrophil behavior once taken up; contain inflammatory cytokines and are vital to oncogenic signaling; prothrombotic	Released from tumor cells

Although NADPH oxidase inhibition has been shown to prevent NETosis, not all NETosis appears to be ROS-dependent. In fact, the mechanism of NET release appears to be influenced by the presence or absence of ROS [3, 49]. ROS-dependent NETosis results in neutrophil cell death, known as lytic NETosis, wherein the cell membrane lyses and decondensed chromatin forms NETs. In contrast, ROS-independent NETosis is much more rapid, taking minutes as opposed to hours. The nuclear envelope disintegrates, and the decondensed chromatin is extruded as NETs via vesicular transport; the preserved integrity of the plasma membrane allows the anuclear neutrophil to survive and retain functionality. Though mechanisms leading to each process and their distinct effects remain unclear, vital NETosis appears more commonly in the context of infectious disease, whereas lytic NETosis is observed in sterile injury [7, 49]. Further investigation of whether NETosis is vital, lytic, or both in the context of cancer is needed.

Regardless of the stimuli present, certain factors within the neutrophil have been shown to be critical to NET release. These include protein arginine deiminase 4 (PAD4), neutrophil elastase (NE), and myeloperoxidase (MPO). PAD4 is a calcium-dependent enzyme localized within the nucleus, cytoplasm, and secretory granules of neutrophils. Inside the nuclear envelope, PAD4 catalyzes hypercitrullination of histones H3, H2A, and H4, which contributes to chromatin decondensation [50]. Histone citrullination is widely considered to be characteristic of NETosis and fluorescent antibodies against citrullinated histones are often used to identify released NETs [38, 51]. Selective inhibition of PAD4 has been shown to abrogate NETosis in response to PMA and a wide variety of physiological stimuli, supporting the critical role of PAD4 in NET release [39]. However, NETosis has been observed in the absence of either or both histone citrullination and PAD4 activation, which suggests additional mechanisms for NET release. Jorch and Kubes’s [7] recent review proposes that other neutrophil granule components, such as NE and MPO, may be sufficient for PAD-independent NETosis. NE is capable of cleaving histones within the nuclear envelope to begin chromatin decondensation. Although MPO independently also appears to have a modest effect on decondensation, its contribution to altering the chromatin structure increases in the presence of NE. MPO binds to DNA and catalyzes oxidative reactions, which promotes the relocation of NE from the cytoplasm to the nucleus [52]. Furthermore, NE and MPO have both been observed to decorate the DNA backbone of NET fibers [2].

Toll-like receptor 4 (TLR4), a receptor triggered by microbial components mostly expressed on surveilling immune cells including the neutrophil cell membrane, is capable of stimulating NETosis via a protein called high mobility group box 1 (HMGB1) both in vitro and in vivo in mice. Tadie et al. [33] incubated wild type and TLR4-deficient mouse neutrophils with HMGB1 and discovered that TLR4-deficient neutrophils released significantly less DNA and citrullinated histone 3 (citH3) than wild type neutrophils. Furthermore, an NADPH oxidase inhibitor was sufficient to prevent NETosis via PMA stimulation, but did not reduce NETosis via HMGB1, suggesting that HMGB1 mediates NETosis via a ROS-independent pathway. Additionally, HMGB1 can also bind to LPS, creating a synergistic effect promoting NETosis. The authors found that pretreating mice with both LPS and HMGB1 increased in vitro NETosis of neutrophils harvested upon sacrifice when compared to LPS alone. Upon treatment with LPS and HMGB1 antibodies, NETosis was diminished. Interestingly, HMGB1 is overexpressed in several cancers, including the human breast cancer cell line MCF-7, in which its silencing provoked significantly higher levels of tumor cell apoptosis and lower levels of migration and invasion in in vitro assays [30]. To what extent these anti-tumoral effects occur due to the disruption of HMGB1-induced NETosis merits further investigation.

The receptor for advanced glycation end products (RAGE) is another damage-associated molecular pattern (DAMP) receptor that plays a critical role in the pathogenesis of breast cancer [53] and has also been implicated in NET formation [54]. Neutrophils collected from RAGE null mice have diminished potential for NETosis as well as reduced intra-tumoral and circulating NET biomarkers. RAGE has been implicated as a key inducer of autophagy [55], a cell survival mechanism which has also been associated with NET formation [56, 57]. Neutrophils undergoing NET formation show upregulated autophagy [54, 58]. Furthermore, pharmacologic inhibition of autophagy prevents NETs from forming [59]. While the precise mechanism for autophagy-induced NET formation remains unclear, this is an area of active study.

Neutrophil maturity may also affect capacity for NETosis. Terminally differentiated neutrophils may undergo NETosis as a result of the reactivation of cyclin-dependent kinase 6; knockout or inhibition of cyclin-dependent kinase 6 produces neutrophils with a reduced capacity for NETosis [60]. Additionally, granular content may differ between mature and immature neutrophils, as neutrophils derived from acute myeloid leukemia patients, which contain markers associated with neutrophil immaturity, show a reduced capacity for NET formation when challenged with PMA [61]. Mackey et al.’s [62] recent review details the role of neutrophil maturity in the context of cancer in greater detail.

**Fig. 1:**
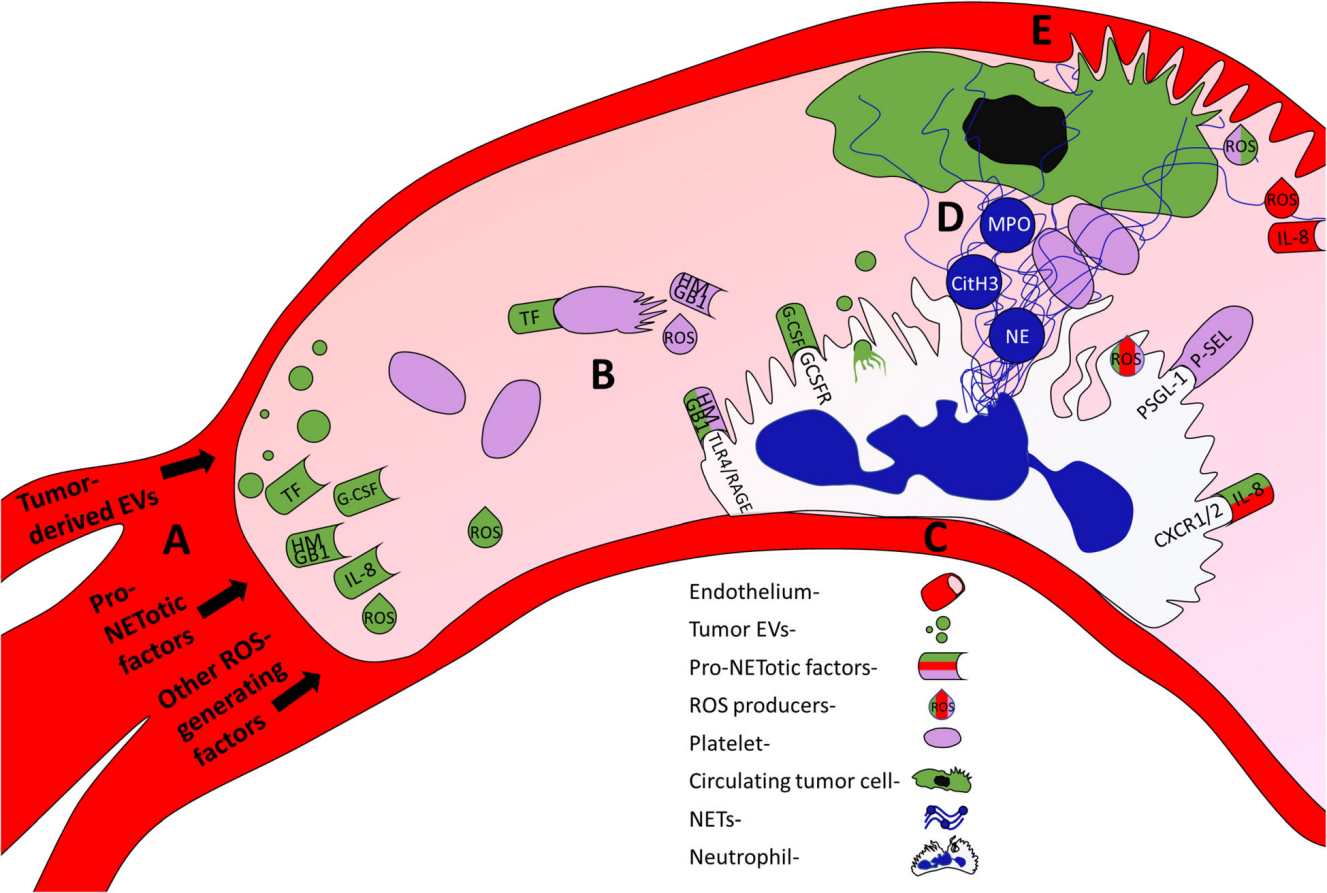
Multicellular interactions between neutrophils, tumor cells, platelets and endothelial cells result in NETosis. Shapes are color coded by their cell or granule of origin: neutrophil nucleus and NETs (dark blue), tumor (green), platelet (lavender), and endothelium (red). (**a**) The primary tumor site releases extracellular vesicles (EVs), various ROS generating proinflammatory factors (indicated by “ROS”), and specific pro-NETotic factors into the circulation. (**b**) Tissue factor (TF) released from tumor cells activates platelets, provoking the release of HMGB1 and further ROS generation. Compounding this, tumor released proinflammatory factors may provoke the endothelium as well, dependent on tumor phenotype. (**c**) Factors released from the tumor, activated platelets, and activated endothelium bind to their respective receptors on the neutrophil, causing NET release. Activated platelets can also directly bind to the neutrophil surface through P-selectin (P-SEL)/P-selectin glycoprotein ligand 1 (PSGL-1) interactions to generate NETosis. Furthermore, tumor-derived EVs may also promote NET release through neutrophil phagocytosis of the tumor membrane fragments and encapsulated factors. The neutrophil flattens and adheres to the endothelium during this process. (**d**) Released NETs are decorated with citH3, NE, and MPO and further activate and entrap platelets, leading to the potential for venous thromboembolism. NETs may also capture circulating tumor cells, promoting the formation of metastases. (**e**) NETs damage endothelial cells via proteolytic components such as NE and MPO, causing the release of inflammatory factors, including IL-8, which can further promote NET release and neutrophil recruitment. Arrested tumor cells further damage endothelial cells as they extravasate. The now highly inflammatory, crowded environment forms the pre-metastatic niche.

Figure 1 illustrates the role of ROS, neutrophil granule enzymes MPO and NE, citH3, and neutrophil surface receptors in promoting tumor-derived NETosis. The following sections will highlight the key cellular interactions between neutrophils, tumor cells, endothelial cells, and platelets to enable NET release, which are also displayed in Fig. 1.

#### Tumor cells

Cancer cells prime neutrophils towards a pro-NETotic phenotype via the expression and release of various pro-NETotic factors such as granulocyte-colony-stimulating factor (G-CSF) and interleukin-8 (IL-8). Tumor cells have been observed to act as a source of overexpressed G-CSF in both murine and human tumors, which activates neutrophils via binding to the G-CSF receptor on the cell surface [63–65]. The normal function of G-CSF is to regulate leukocyte differentiation, maturation, survival, and proliferation, as well as facilitate their translocation from the bone marrow to the bloodstream. Overexpression of G-CSF in cancer, however, can result in an overabundance of neutrophils in the blood, ROS generation in neutrophils, and subsequent NETosis [19, 36]. Cedervall et al. [37] have shown that inhibition of G-CSF function in the MMTV-PyMT transgenic mouse mammary carcinoma model reduced NETosis and improved peripheral blood flow. In this study, MMTV-PyMT mice were injected daily with an anti-G-CSF antibody. After 7 days of treatment, the perfused vessel area within renal tissue was measured by perfusing the mice with FITC conjugated lectin before sacrifice and cryosectioning. Mice that received anti-G-CSF treatment exhibited significantly increased fluorescent perfusion due to the reduced NET formation. Ultimately, increased G-CSF expression results in enhanced metastatic potential for a variety of cancers, including breast cancer, by activating neutrophils in the pre-metastatic niche [65, 66].

Neutrophils are chemotactically attracted to tumor cells through secretion of IL-8 (also known as CXCL8). It should be noted that human IL-8 does not have a direct counterpart in mice. IL-8 binds to G-protein coupled receptors, CXCR1 and CXCR2, which are expressed by neutrophils [67]. IL-8 plays an important role in recruiting neutrophils to sites of inflammation; as such, women with breast cancer have higher serum levels of IL-8 compared to healthy patients. Additionally, IL-8 levels strongly correlate with disease progression [68]. In infectious disease, recruitment towards inflammation may be beneficial, as Xu et al. [69] have shown that reduced CXCR1 and CXCR2 expression on neutrophils correlated with negative clinical outcomes in hepatitis B due to insufficient neutrophil recruitment. Other studies have confirmed that inhibition of IL-8 receptors prevents human neutrophil chemotaxis in vitro [70]. IL-8 production in multiple cancer types, including breast cancer, has also been associated with increased metastatic potential [35]. IL-8 is capable of stimulating NETosis in human neutrophils in vitro, and the addition of IL-8 antibodies abolishes this effect [5, 34]. In mice, CXCL1 (KC), CXCL2 (MIP-2), and CXCL5 and 6 (LIX) serve as functional homologs of IL-8 promoting murine neutrophil chemotaxis; KC and MIP-2 bind with CXCR2. While the roles of MIP-2 and LIX in NETosis are unclear, KC has been shown to promote NETosis in murine sepsis models [71, 72].

Finally, while PAD4 is localized within the nucleus, cytoplasm, and secretory granules of neutrophils, it has also been shown to be expressed in multiple tumor cell lines. Chang et al. [40] showed that breast tumors in particular had the greatest PAD4 expression in a variety of human malignancies, including lung adenocarcinomas, colorectal adenocarcinomas, renal cancer cells, and others; additionally, elevated levels of PAD4 were detected in patient plasma and associated with the presence of other tumor biomarkers. The mechanism concerning how PAD4 is exported from tumor cells and whether extracellular PAD4 can stimulate NETosis has not been previously studied.

#### Endothelial cells

In addition to being secreted by tumor cells, IL-8 is also known to be produced via endothelial cell (EC) activation [73]. EC activation occurs when the vasculature is exposed to oxidative stress via injury, inflammation, chemotherapy, or ionizing radiation [74]. Activated ECs release inflammatory cytokines and growth factors and also express several adhesion molecules on their surface such as P-selectin, E-selectin, and ICAM-1 to facilitate neutrophil rolling, adhesion, and transmigration to the inflamed site [75]. Gupta et al. [34] investigated the role of ECs in promoting NETosis and found that activated ECs co-cultured with neutrophils in vitro resulted in NET formation that is partially mediated by IL-8. Released NETs exposed to the surface of ECs for prolonged time periods (18 h of neutrophil-EC co-culture) resulted in eventual EC injury and death, which could be inhibited through NET dissolution by a DNA-degrading enzyme, DNase I. NET-induced EC injury and death has also been observed in vivo, though this has been demonstrated indirectly. Schreiber et al. [76] found that DNase I treatment reduced NET formation and protected mice from blood vessel inflammation, known as vasculitis. Additionally, Knight et al. [77] showed that PAD4 inhibition via daily injections of Cl-amidine was effective in reducing NETosis in mice, as well as preventing further vascular damage and atherosclerosis. Little research has been done to elucidate the link between NETosis and cancer-induced endothelial damage. However, tumor cells themselves can contribute to EC inflammation, which can enhance the potential to induce NETosis by further increasing EC damage [34, 78]. The link between cancer-associated EC activation and NETosis may be worth further investigation; however, since many of the same stimuli provoke both neutrophil and EC response, establishing causality may be difficult.

#### Platelets

Activated platelets also stimulate NETosis, which sets up a positive feedback loop, as released NETs are known to strongly promote a prothrombotic state that further enhances platelet activation [79]. Much like endothelial cells, platelets must undergo activation prior to stimulating NETosis [25, 31]. Many tumor cell lines including certain breast cancers have been shown to overexpress and release tissue factor (TF) [44], which is a well-established platelet activator. TF levels have been shown to correlate with mortality in breast cancer patients [80]. However, the use of TF as a biomarker for specifically defining VTE risk has been demonstrated for some cancers yet remains inconclusive for others [81]. Nevertheless, Razak et al. [82] suggest that cancer may activate platelets through uptake of small tumor-derived extracellular vesicles, which often contain TF. Neutrophils also contain tissue factor, which is released from NETs to further promote a positive feedback loop by stimulating platelets [42, 43]. Further investigation into the mechanisms of TF-mediated increases in mortality independent of VTE risk would be interesting.

Post activation, platelets can stimulate NET release through direct adhesive interactions with neutrophils [41, 83]; upon activation, platelets rapidly translocate an adhesion molecule known as P-selectin to their surface [84], which can bind to the neutrophil surface receptor P-selectin glycoprotein ligand-1 (PSGL-1) to promote neutrophil-platelet adhesion [85], neutrophil activation [86], and subsequent NET release. Etulain et al. [41] show thrombin activated platelets elicit NETosis both in vitro and in vivo in murine neutrophils, and NET formation does not occur when either P-selectin or PSGL-1 inhibitory antibodies are introduced. NETosis was also abolished in P-selectin knockout mice. Interestingly, solubilized P-selectin alone was also observed to stimulate NETosis, but to a lesser extent than activated platelets [41]. This potential NETosis pathway could also be relevant in cancer where high levels of soluble P-selectin found in patient blood plasma have been linked to higher rates of VTE [87].

Both TLR4 and HMGB1 are also expressed by platelets and have been shown to be another means of platelet-stimulated NETosis relevant to cancer [25, 31]. In septic mice, Clark et al. [31] were the first to show that LPS binds to TLR4 to enable platelet activation, neutrophil-platelet aggregate formation, subsequent neutrophil activation, and NET release. Platelet HMGB1 can cause NETosis through neutrophil TLR4 activation, or alternatively can bind to the neutrophil RAGE receptor to stimulate NETosis. Maugeri et al. [25] found that when human platelets were activated with a variety of factors, including thrombin or collagen, they were able to stimulate NETosis via HMGB1. NETosis was abolished when RAGE was blocked via antibodies. The authors also show that HMGB1 is no longer present in platelets post activation, indicating that it is released rather than translocated to the membrane. It is conceivable that platelets may serve as an intermediary between tumor cells to influence neutrophils and promote NETosis via the release of platelet-activating soluble factors, such as HMGB1.

#### Extracellular vesicles

Though initially thought to solely be biomarkers, current literature suggests that extracellular vesicles (EVs) actively contribute to angiogenesis, metastasis, and coagulation [21, 88]. The role of EVs in promoting NETosis in the context of cancer is only just being explored. Broadly, EVs are formed when a piece of membrane sheds from the parent cell to form membrane-enclosed particles, the contents of which depend on the phenotype of the parent cell. Ultimately, any cytoplasmic material in the parent cell can be present in its EVs; EVs are extremely heterogenous and can also form from the Golgi or endosomal membrane [89]. Though EVs can be further subcategorized based on size or origin, the term “extracellular vesicle” refers to any particle 50–1500 nm in diameter [90]. EV release often occurs as a stress response. Consequently, EVs are more highly concentrated in cancer patients than in healthy individuals. Elevated EV content in breast cancer patient blood serves as an indicator of more advanced disease stage and is associated with worse therapeutic success and lower 3-year survival rates [91]. While the cargo, RNA, DNA, and membrane proteins present in EVs from cancer patients have not yet been fully characterized, cancer-derived EVs have been associated with high expression of pro-NETotic and pro-tumoral factors such as interleukins and G-CSF [92–94]. We will discuss EVs derived from tumor-burdened organisms and from tumor cell culture. As tumor-derived EVs are just recently being observed to modulate neutrophil behavior, including NETosis, it is not surprising that the growth factors and cytokines these EVs carry can further contribute to the inflammatory microenvironment of a nascent pre-metastatic niche.

Leal et al.’s recent study [21] shows that EVs derived from cultured 4T1 mouse breast cancer cells stimulated NETosis in vitro in neutrophils primed with G-CSF. BALB/c mice with orthotopic mammary 4T1 tumors were shown to have significantly more EVs present in blood plasma compared to control mice without tumors. The evaluated population contained particles approximately 80–110 nm in diameter. Mice containing 4T1 tumors exhibited more rapid coagulation in venous and arterial injury models compared to control mice. The enhanced prothrombotic state of 4T1 mice could be inhibited through use of DNase I, suggesting a role of NETs in platelet activation. Notably, healthy mice injected with G-CSF and culture-derived 4T1 EVs experienced more rapid coagulation induced via photochemical vascular injury than did healthy mice given G-CSF only. NETs were observed (though not quantified) within these thrombi, suggesting that EVs could lead to NET release and subsequent coagulation in vivo. However, the use of exclusively tumor-derived EVs is limiting, as it does not account for the release and content of EVs derived from other blood cells in tumor-burdened organisms. EVs released from other cells such as platelets, endothelial cells, and macrophages may also be tumor mediated, since EVs facilitate intracellular communication between tumors and other cells [92]. Despite this, to our knowledge, Leal et al.’s study has been the only published work to examine the direct stimulatory effect of tumor-derived EVs on NETosis.

Similarly, the specific mechanisms of interaction between neutrophils and EVs leading to NETosis are largely unknown. However, Headley et al. [95] utilized fluorescence intravital microscopy of lungs in live mice to show that B16 melanoma cells, injected intravascularly via the tail vein, attached to the pulmonary endothelium and subsequently released large membrane bound particles of around 5 μm. Fascinatingly, the authors observed that neutrophils and other immune cells had phagocytosed fragments of these tumor-derived microparticles in vivo. As such, it is not unreasonable to conclude that ingested tumor material may have a stimulatory effect on immune cells. These implications are supported by evidence showing that neutrophils uptake tumor-derived DNA delivered via EVs, which may contain pro-NETotic cargo. In fact, Chennakrishnaiah et al. [96] recently showed that white blood cells contained the highest concentration of human epidermal growth factor receptor 2 (HER2) oncogenic DNA in SCID mice bearing BT474 breast tumor xenografts (a HER2-positive human breast carcinoma) compared to other blood components, including plasma, suggesting that neutrophils may be especially prone to stimulation from tumor-derived EVs. A parallel experiment examining the oncogenic DNA content of a different human breast cancer oncogene, HRAS, within the white blood cells of RAS-3 burdened SCID mice showed that neutrophils were the major contributor to this uptake and that neutrophil depletion resulted in far higher plasma oncogenic DNA concentration. Finally, RAS-3-derived exosomes were shown to trigger a significant increase in endogenous expression of IL-8 in vitro in human neutrophil-like cells, or HL60. These findings provide interesting insights into the NETosis stimulation exhibited by tumor-derived EVs. NETosis may be both directly induced via stimulants expressed by the tumor cell and contained within EVs, and EVs may induce neutrophils to produce their own NETosis stimulants. However, our understanding of the role of EVs in causing NETosis remains limited. Though proteomic analysis has been performed on a variety of tumor-derived EV populations, the content of known NETotic agents has not been examined. Additionally, whether neutrophils internalize EVs predominantly through phagocytosis or receptor mediated endocytosis is also unknown.

### Impact of NETosis on VTE and metastasis

**Fig. 2:**
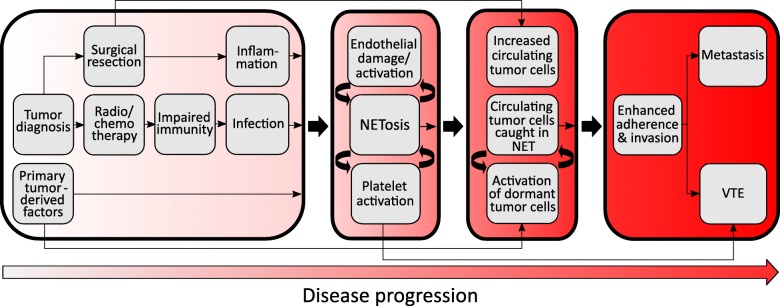
Flowchart illustrating the causes of NET formation and the means by which NETosis leads to disease progression downstream

Figure 2 shows the integration of multiple NET stimuli and the downstream effects of NET release including enhanced VTE and metastasis. NETs have been identified as a prognostic indicator of VTE and are at least partially responsible for the hypercoagulable state observed in cancer patients. It is estimated that women with breast cancer are three to four times more likely to develop VTE compared to age-matched women without breast cancer [97]. VTE occurring in breast cancer patients has also been linked to reduced patient survival and tumor recurrence. Mauracher et al. [26] recently observed that high plasma levels of NET marker, citH3, were predictive of an increased risk of VTE for 2 years post diagnosis or relapse in a cohort of nearly a thousand cancer patients; interestingly, levels of circulating DNA were only predictive of increased VTE risk during the first 3 to 6 months. Of the tumor sites examined, brain, lung, and breast showed the highest frequency of patients whose sera contained elevated citH3 levels. For these patients, the 2-year risk of VTE was 14.5%, as opposed to 8.5% for patients lacking elevated citH3. An increase in citH3 of only 100 ng/mL was found to translate to a 13% higher risk of VTE, suggesting that even mild NETosis may severely impact prognosis. The hypercoagulability characteristic of cancer patients has been shown to be largely NETosis dependent in breast cancer models [21]. Demers et al. [19] showed that G-CSF seems to further drive the prothrombotic state by priming neutrophils for NETosis in a 4T1 mammary carcinoma mouse model. Tumor-bearing mice experienced a significant decrease in both platelet and neutrophil counts consistent with thrombus formation and had reduced tail bleed times. In tumor-burdened mice, the highest levels of citH3 were present in the later stages of disease.

It has also been suggested that NETs promote the formation of the pre-metastatic niche, at least partly by entrapping circulating tumor cells in their web-like structure, providing a site from which these cells may then extravasate. Cools-Lartigue et al. [10] showed via intravital microscopy that NETs were able to arrest circulating tumor cells in the pulmonary and hepatic microvasculature. A septic state was induced in C57BL/6 J mice prior to intrasplenic injection of H59 Lewis lung carcinoma cells. Micrometastases were observed within 48 h, with both NE inhibitor and DNase I treatment abolishing this effect; non-septic mice showed few micrometastases, suggesting that pro-NETotic stimuli are required to enhance NET-mediated metastasis. Park et al.’s [27] recent study shows that 4T1 breast cancer cells injected into the tail vein of LysM-EGFP mice were found within lungs and caused NET formation; immunofluorescence staining of lung tissue sections showed via DNA and NE fluorescence that tumor cells were sufficient to provoke increased rates of lytic NETosis for up to 4 days post injection. DNase I-coated nanoparticle treatment reduced tumor invasion in vitro and the number and size of lung metastases in vivo.

Interestingly, surgical resection aimed at decreasing tumor burden can actually promote metastasis through neutrophil activation and subsequent NET formation. Increased NETosis in patients undergoing liver resection due to metastatic colorectal cancer correlates to markedly lower cancer-free survival [14]. This effect of increased metastasis following surgical stress was reproduced in mouse models and was abolished by DNase I treatment or inhibiting PAD4 to dissociate NETs or prevent their release, respectively. Neutrophil HMGB1 release occurred concurrently with pro-NETotic stimulation, and HMGB1 was also associated with NETs. This represents a means by which NETs may directly activate platelets and other neutrophils, eventually forming a thrombus. Circulating tumor cells (the presence of which may be increased by surgical disruption of the primary tumor) could then be captured due to partial vessel blockage and the coagulating microenvironment around the NET. Simultaneously, the capacity of NETs to damage endothelial cells likely enables arrested tumor cells to adhere to the activated endothelium, eventually extravasating and establishing a new metastatic site. Interestingly, NETs have also been shown to activate dormant single breast tumor cells in mouse lungs, which can then lead to metastasis development. Cancer cell activation from dormancy is thought to occur via the remodeling of extracellular matrix due to NET-associated NE and is further facilitated by G-CSF [13].

It is reasonable to conclude that tumor-driven NETosis alone, even without surgical stress or major infection, can also serve to drive metastasis. Pro-NETotic factors are known to be overexpressed by many tumor lines, and multiple murine breast cancer models have been shown to promote NETosis. However, much remains unclear about the specific mechanism in which cancer promotes metastasis through NET formation. It is currently unknown whether NETs predominantly contribute to metastatic establishment via endothelial damage or direct sequestration of tumor cells. Additionally, little is known about the timeline of NET generation. It is possible that the primary tumor site must reach enough development to elicit NETosis, which then promotes the establishment of metastases. Alternatively, pioneering tumor cells may secrete pro-NETotic factors which then provoke a NET-induced inflammatory state from surrounding neutrophils, favoring tumor cell invasion and further sequestration of circulating tumor cells.

### NETs as biomarkers and clinical targets

The ability to detect NETs would likely be of significant prognostic use in differentiating patients at higher risk of metastatic progression or VTE, thereby enabling clinicians to better personalize treatment regimens. To develop a clinical screening tool for NETs, a standardized definition of “normal” levels of NETosis would need to be established and has not yet been presented in the literature. The simplest means of in vivo NET detection involves measurement of NET-associated products in the blood such as circulating cell-free DNA, citH3, NE, and MPO. For example, free circulating DNA has been quantified in both colorectal and breast cancer patient serum samples via a simple nucleic acid staining assay [98, 99]. However, even though circulating DNA is known to correlate with breast tumor size and malignancy [100], it lacks specificity in measuring NETosis. An increased amount of DNA in cancer patient serum can also be attributable to other factors such as apoptotic and necrotic cells. Measuring circulating MPO/DNA conjugates is more specific for NET formation than evaluation of cell-free DNA alone [101]. Citrullinated histone H3 (citH3) is formed as a result of PAD4-mediated citrullination during NET formation and represents the most specific biomarker for circulating NETs [26]. In addition, citH3 may be of prognostic significance, as Thålin et al. [102] observed that high plasma content of citH3 was a significant indicator of short-term mortality in late-stage cancer patients, even when compared to severely ill patients without cancer. Additionally, IL-8 levels were found to correlate with levels of citH3. Since higher levels of IL-8 would result in increased neutrophil recruitment, it would be reasonable to conclude that this higher density of neutrophils would subsequently lead to increased NETosis. Despite this, other markers associated with NETs including NE and MPO were not found to differ significantly between severely ill patients with and without malignancy; however, these neutrophil-derived enzymes can be independently released during neutrophil degranulation in the absence of NET formation, and therefore may not be reliable NET-specific biomarkers. Indeed, citH3 seems to be the most consistent indicator of NETosis. While levels of other markers may provide useful insight into neutrophil behavior, citH3 is highly specific to NETosis and thus would be valuable in understanding variances between other NET-associated biomarkers. CitH3 levels are also predictive of VTE risk in newly diagnosed patients, further supporting its diagnostic utility [26].

The development of clinical therapies specifically targeting NETs in cancer is in its infancy. Inhibition of NETosis has been achieved through several means, though these vary in their potential for clinical therapies. For instance, DNase I treatment degrades NETs and results in a loss of the web-like structure and a reduction in the capacity to promote metastasis in several studies [10, 14, 21, 34]. In addition, DNase I has been shown to decrease tumor volume in rats when injected intramuscularly or intraperitoneally in conjunction with other proteases (papain, trypsin, and chymotrypsin) [103]; however, it is not known whether these effects are due primarily to NET inhibition. Currently, DNase I is used clinically in the treatment of cystic fibrosis, as it decreases the NETosis-mediated buildup of mucous viscosity, resulting in improved lung function [6]. However, in this context, DNase I is delivered via nebulizer, which would likely be ineffectual in most cancer treatments, though it would be fascinating to observe whether nebulized DNase I would have a preventative effect on lung metastasis. Additionally, DNase I injection may have off-target effects, including compromising the immunoprotective function of NETs.

Inhibition of components integral to NETosis, such as NE or PAD4, would likely have similar off-target effects due to their involvement in other key pathways, potentially disrupting normal neutrophil function. Small molecule inhibitors of PAD4 for NET inhibition are under active investigation and include Cl-amidine and F-amidine, irreversible inhibitors that inactivate calcium-bound PAD4 [104]. However, these lack specificity and interact with other PAD-family enzymes. Recently, Lewis et al. [105] synthesized two reversible inhibitors which overcome this hurdle, GSK199 and GSK484, both of which exhibit high specificity for PAD4 and inhibit NETosis in both mouse and human neutrophils. GSK484 was recently shown to prevent tumor-associated renal dysfunction in mice, which was determined to be NET-mediated; the inhibitory effects of GSK484 were as effective as DNase I [106]. Additionally, a recent study by Yazdani et al. [107] indicates that PAD4-knockout mice challenged with subcutaneous tumor injection of colorectal and hepatocellular carcinoma tumor cells experienced slower tumor growth and smaller metastases similar to mice treated with daily DNase I injection. NETs were not observable in excised tumor tissue in PAD4-knockout mice. Finally, the authors showed that NETosis at the primary tumor site may contribute to tumor cell survival through enhanced mitochondrial biogenesis. This data further supports the need to develop NET-targeting treatments, as these would be of great therapeutic benefit in both the context of the primary tumor site and the pre-metastatic niche.

Efforts targeting cell adhesive molecules, such as P-selectin, could also prove problematic. Though successful results of a stage II clinical trial for the use of the P-selectin inhibitor crizanlizumab in sickle cell anemia to prevent vaso-occlusion were recently published [108], it would be reasonable to conclude that such a therapy may interfere with leukocyte function. Though P-selectin and PSGL-1 antibodies have been shown to inhibit NETosis in mice [41], the disruption of leukocyte adhesion molecule binding capacity could decrease neutrophil recruitment in response to infection in cancer patients already suffering from an immunocompromised state. Off-target effects could potentially be mitigated via the development of new, more specific delivery vehicles, such as functionalized, targeted nanoparticles.

Alternatively, the adaptation of FDA-approved drugs could facilitate the development of effective anti-NET treatments. For instance, the inhibitory effect of aspirin on NETs has yielded some promising results in animal models. Lapponi et al. [109] showed that aspirin prevented NET-induced injury of the lung endothelium by inhibiting platelet activation and subsequent NET formation in mice. The inhibitory effect of aspirin on NF-κB, an inflammatory transcriptional regulator that plays a role in some pathways promoting NETosis, was also demonstrated. The authors found that aspirin treatment effectively inhibited NETs in human neutrophils in vitro and resulted in higher bacteria counts in infection-burdened mice in vivo, suggesting a loss of normal NET functionality. There is evidence to support the use of aspirin in clinical treatment. In one meta-analysis, patients using aspirin daily had significantly reduced mortality and risk of distant metastases for adenocarcinomas. Interestingly, this effect did not appear to be dose dependent [110]. Aspirin has also been shown to be effective in reducing metastasis in patients suffering from breast cancer specifically [111].

Another FDA-approved drug, hydroxychloroquine, originally used to treat malaria, has been shown to inhibit NETosis [17, 54, 112]. While the mechanism behind NET inhibition by hydroxychloroquine is unclear, it may be related to autophagy inhibition [113]. However, a phase II clinical study on patients with advanced pancreatic cancer produced little clinical effect. The authors do suggest, however, that combination therapy may prove more effective [114]. Furthermore, use of hydroxychloroquine as a neoadjuvant treatment in earlier stage disease holds significant promise [115]. Remarkably, and perhaps not coincidentally, hydroxychloroquine also inhibits leukocyte phagocytosis [116]. Thus, it may be possible that hydroxychloroquine could inhibit neutrophil uptake of tumor-derived EVs, thus reducing NETosis. However, the precise mechanism by which this uptake occurs is unknown, as are the mechanisms behind tumor-derived EV stimulated NETosis. Due to the associated complications of NETs including increased VTE risk and metastasis, which are both negatively associated with breast cancer patient outcome, it is crucial for future research efforts to focus on further investigation of new specific targets to prevent NET formation.

## Conclusion

Evidence is mounting that NETs play a significant detrimental role in the inflammatory state of cancer. We have presented several classical NETotic stimuli, as well as stimuli that have been implicitly or explicitly demonstrated to induce NETosis specifically within the context of cancer, though the mechanisms by which such stimuli occur are not yet entirely defined. We have also discussed the negative outcomes NETs promote and have highlighted potential NET-specific targets to investigate and utilize to develop therapies for clinical translation. The next vital step will be untangling the web of crosstalk between neutrophils, tumor cells, endothelial cells, platelets, and extracellular vesicles, and eventually the influence of other components of the innate and adaptive immune systems on cancer progression. Better understanding of these processes will enable the development of precise NET-targeted therapies and diagnostic tools, potentially allowing the identification of tumors with the potential for metastasis, earlier diagnosis, and more personalized and effective treatments for breast cancer patients.

## Data Availability

Not applicable.

## Abbreviations

CitH3: Citrullinated histone 3
DAMP: Damage-associated molecular pattern
EC: Endothelial cell
EV: Extracellular vesicle
G-CSF: Granulocyte-colony-stimulating factor
HER2: Human epidermal growth factor 2
HMGB1: High mobility group box 1
IL-8: Interleukin-8
LPS: Lipopolysaccharide
MPO: Myeloperoxidase
NE: Neutrophil elastase
NET: Neutrophil extracellular trap
PAD4: Protein arginine deiminase 4
PMA: Phorbol 12-myristate 13-acetate
P-SEL: P-selectin
PSGL-1: P-selectin glycoprotein ligand-1
RAGE: Receptor for advanced glycation end products
ROS: Reactive oxygen species
TF: Tissue factor
TLR4: Toll-like receptor 4
VTE: Venous thromboembolism
